# Adenosine-induced stress myocardial perfusion MRI using SW-CG-HYPR with whole left ventricular coverage: comparison of results with X-ray angiography in patients with suspected CAD

**DOI:** 10.1186/1532-429X-13-S1-P78

**Published:** 2011-02-02

**Authors:** Heng Ma, Lan Ge, Jing An, Lixin Jin, Renate Jerecic, Kuncheng Li, Debiao Li

**Affiliations:** 1 Xuanwu Hospital, Capital Medical University, Beijing, China; 2 Department of Radiology, Northwestern University, Chicago, IL, USA; 3 Siemens Healthcare, MR Collaboration NE Asia, Siemens Mindit Magnetic Resonance, Shenzhen, China; 4 Siemens Healthcare, MR Collaboration NE Asia, Siemens Limited China, Shanghai, China

## Introduction

Myocardial perfusion MRI with sliding-window conjugate-gradient HYPR (SW-CG-HYPR) allows increased spatial coverage (whole left ventricular coverage), resolution, signal-to-noise ratio and reduced motion artifacts. The accuracy of this technique for detecting coronary artery disease (CAD) has not been determined in a large number of patients.

## Purpose

The purpose of this study was to prospectively evaluate the diagnostic performance of adenosine-induced stress myocardial perfusion MRI with SW-CG-HYPR in patients with suspected CAD.

## Methods

Forty consecutive patients (23 men and 17 women; mean age, 56 ± 15 years) with suspected CAD who were scheduled for coronary angiography underwent myocardial adenosine stress perfusion MRI with SW-CG-HYPR at 3.0T. Perfusion defects were interpreted visually by 2 blinded observers and were correlated to x-ray angiographic stenoses ≥ 50%.

## Results

**Figure 1:**
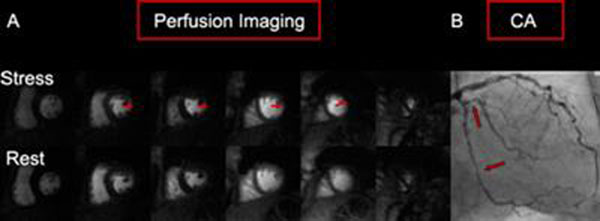
A 56-year-old man with no prior cardiac history who presented with chest pain. **(A)** Myocardial perfusion MRI with sliding-window conjugate-gradient HYPR (SW-CG-HYPR) detects perfusion defects in the basal, mid, and apical lateral segments, corresponding to significant stenoses in the left circumflex coronary artery (LCX). **(B)** Coronary angiography (CA) confirms significant stenoses in the LCX.

The prevalence of CAD was 55%. In the per-patient analysis, the sensitivity, specificity and accuracy of SW-CG-HYPR myocardial perfusion imaging were 95%, 83% and 90%, respectively. In the per-vessel analysis, these values were 98%, 89% and 93%, respectively. Figure 1 illustrates the detection of significant CAD by SW-CG-HYPR myocardial perfusion imaging with correlation to X-ray coronary angiography.

## Conclusions

Adenosine-Induced stress myocardial perfusion MRI using SW-CG-HYPR allows whole left ventricular coverage and has high diagnostic accuracy in patients with suspected CAD.

